# Editorial: Investigating AI-based smart precision agriculture techniques

**DOI:** 10.3389/fpls.2023.1237783

**Published:** 2023-07-11

**Authors:** Uzair Aslam Bhatti, Mehdi Masud, Sibghat Ullah Bazai, Hao Tang

**Affiliations:** ^1^School of Information and Communication Engineering, Hainan University, Haikou, China; ^2^Department of Computer Science, Taif University, Taif, Saudi Arabia; ^3^Balochistan University of Information Technology, Engineering, and Management Sciences (BUITEMS), Quetta, Pakistan

**Keywords:** agriculture, image processing, deep learning, remote sensing, data science

## Overview

The monsoon plays a pivotal role in determining agricultural output. The success of crops planted during a monsoon season is highly contingent on the prevailing weather conditions. Access to real-time meteorological information is crucial for farmers to make informed decisions regarding crop management, thereby reducing the risks and losses associated with adverse climatic conditions.

Agricultural fields are typically managed on a field-by-field basis, without considering the spatial and temporal variability of the soil. This approach can lead to uninformed decisions by farmers regarding inputs such as fertilizers, irrigation systems, and labor, resulting in suboptimal harvests. To address this, precision agriculture aims to optimize resource utilization by effectively managing the temporal and geographical variability of soil and ecosystem conditions. The advent of the Internet of Things (IoT) and sensor-edge connecting devices has greatly facilitated the collection of agricultural data in a smart manner for farmers.

In addition to weather-related challenges, economic difficulties also impact farming practices and productivity, particularly in rural and semi-rural areas. Farmers face daily obstacles such as pesticide use, water scarcity, resource limitations, and poor soil quality. Overcoming these challenges requires the strategic determination of best practices and approaches.

Smart precision agriculture emerges as an innovative solution that leverages cutting-edge technology to enhance crop yields sustainably. By integrating smart IoT devices and sensors, farmers can optimize agricultural output while minimizing their field work time. Smart technologies enable more efficient resource usage, including reduced water and power consumption, and constant monitoring of variables like humidity and temperature. Internet of Things-based smart farming utilizes multiple sensors, measuring parameters such as humidity, temperature, and soil moisture, to monitor field conditions effectively.

Despite the potential benefits, Smart Sustainable Agriculture (SSA) faces challenges due to insufficient investment in research and development. Additionally, complex barriers arise from the fragmented nature of agricultural processes, encompassing aspects such as the management and operation of IoT/AI machines, remote sensing, environmental impact assessment, data sharing and management, interoperability, and the analysis and storage of extensive datasets.

The provided text discusses various research papers and studies related to AI-based smart precision agriculture techniques. Here is a summary of each study:

## Chinese agricultural named entity recognition

This study focuses on improving named entity recognition in Chinese agricultural texts, specifically in the context of kiwifruit diseases and pests. The researchers propose a novel model called KIWINER, which incorporates new word detection, an attention-based softlexicon module, and a parallel connection criss-cross attention module. The model achieves high F1-scores on multiple datasets, demonstrating its effectiveness in recognizing kiwifruit-related named entities (Zhang et al.).

## Infrared and visible image fusion in agriculture

The paper presents a distributed fusion architecture called RADFNet for combining infrared and visible images in agricultural applications. The architecture utilizes residual CNN, edge attention, and multiscale channel attention to improve image quality and eliminate environmental interference. Experimental results show that RADFNet outperforms existing image fusion algorithms in terms of visual effect and quantitative metrics (Feng et al.).

## Dome-type planted pumpkin autonomous harvesting framework

This study introduces a framework for autonomous harvesting of dome-type planted pumpkins. The framework includes a keypoint detection method using instance segmentation architecture, combining transformer network and point rendering to address overlapping and improve segmenting precision. Experimental results on a pumpkin image dataset demonstrate the effectiveness of the proposed method in instance segmentation and keypoint detection, with promising application prospects in fruit picking tasks (Yan et al.).

## Genetic diversity analysis of Hopea hainanensis

The research focuses on the genetic diversity of Hopea hainanensis, an endangered tree species found in Hainan Island, China. Using SNP and genotyping-by-sequencing technology, the study analyzes the genetic diversity among different populations of Hopea hainanensis in fragmented habitats. The results reveal low genetic diversity, highlighting the need for genetic diversity research in the conservation of rare and endangered plants (Chen et al.).

## Cassava leaf disease classification

This paper addresses the classification of cassava leaf diseases using deep convolutional neural networks. A multi-scale fusion model based on attention mechanism is proposed to enhance disease feature extraction from cassava leaves. The model achieves improved classification performance compared to the original model, providing support for the recognition and early diagnosis of plant disease leaves (Liu et al.).

## Weed detection in turfgrass

The study focuses on weed detection in turfgrass using deep learning methods. Various convolutional neural networks (DenseNet, EfficientNet-v2, and ResNet) are trained to detect weeds susceptible to herbicides, enabling site-specific weed detection. The results demonstrate high F1 scores and MCC values for most weed species, except for those with similar plant morphology. The proposed method provides an effective strategy for precision herbicide application (Jin et al.).

## Crop rotation and soil health

This research examines the impact of different vegetable cropping systems on soil chemical properties, eggplant photosynthesis, and antioxidant functioning. Leafy vegetable rotation systems are found to significantly improve soil organic matter and available nutrients, as well as enhance eggplant growth and yield. The rotation systems also lead to higher antioxidant enzyme activity, reducing oxidative damage to membranes. The study highlights the benefits of crop rotation for improving the growth and yield of eggplant (Ghani et al.).

## Objective evaluation of turfgrass cultivars

The project addresses the subjectivity in the evaluation of turfgrass cultivars using ordinal data. A model-based approach is proposed to minimize subjectivity and enable objective comparisons of cultivars across different test locations. The model is fitted in a Bayesian framework, allowing the estimation of additional parameters and providing better separation of cultivar means. The approach improves the evaluation procedure and enables more realistic comparisons (Qu et al.).

## Yolo optimization

YOLOv7 maize pests identification method incorporating the Adan optimizer is proposed for the timely and accurate detection of major pests of corn. The study focuses on three major corn pests: corn borer, armyworm, and bollworm. A corn pests dataset is constructed using data augmentation techniques to address the issue of limited pest data. The YOLOv7 network is chosen as the detection model, and the Adan optimizer is introduced to replace the original optimizer for improved efficiency and accuracy while reducing computational costs (Zhang et al.).

## UNET+CBAM disease classification

This research focuses on the identification of apple diseases, specifically Alternaria blotch and brown spot diseases, aiming to improve production efficiency and quality. The paper proposes a disease spot segmentation and disease identification method based on DFL-UNet+CBAM. The primary issues addressed are the low recognition accuracy and poor performance of small spot segmentation in apple leaf disease recognition. The objective is to accurately prevent and control apple diseases, minimize fruit quality degradation, yield reduction, and associated economic losses. The proposed DFL-UNet+CBAM model incorporates a hybrid loss function comprising Dice Loss and Focal Loss (Zhang et al.).

## Pepper leaf segmentation

The study focuses on segmenting pepper leaves from images to aid in the control of pepper leaf diseases. A bidirectional attention fusion network called BAF-Net is proposed (Zhang et al.).

## Author contributions

All authors listed have made a substantial, direct, and intellectual contribution to the work and approved it for publication.

